# No-Fault Compensation Schemes for COVID-19 Vaccines: Best Practice Hallmarks

**DOI:** 10.3389/phrs.2023.1605973

**Published:** 2023-05-03

**Authors:** Duncan Fairgrieve, Marco Rizzi, Claas Kirchhelle, Sam Halabi, Geraint Howells, Normann Witzleb

**Affiliations:** ^1^ British Institute of International and Comparative Law, London, United Kingdom; ^2^ Centre de Recherche Droit Dauphine, Université Paris Dauphine, Paris, France; ^3^ UWA Law School, The University of Western Australia, Perth, WA, Australia; ^4^ School of History, University College Dublin, Dublin, Ireland; ^5^ O’Neill Institute for National and Global Health Law, School of Law, Georgetown University, Washington, DC, United States; ^6^ School of Law, University of Galway, Galway, Ireland; ^7^ Faculty of Law, The Chinese University of Hong Kong, Hong Kong, Hong Kong SAR, China

**Keywords:** vaccine, compensation claims, best practice, COVID-19, injury

The global race for and roll-out of safe and effective COVID-19 vaccines to billions of people is one of the main ways in which the pandemic will be remembered. However, this overwhelmingly positive story of international collaboration and successful vaccine design also contains darker chapters of stark global inequality, vaccine hesitancy, and the way in which (the comparatively few) victims of adverse effects were often failed by governments and existing compensation schemes.

It is now widely accepted that equity and fairness dictate that a swift and effective legal and financial remedy should be available for victims of vaccine injury (1). Vaccination provides both a direct benefit to the person receiving it in terms of personal immunization and an indirect benefit to the broader community as a contribution to wider immunity. Those who suffer serious adverse effects pay a high price for benefits that accrue not only to themselves but to the rest of the population, especially if they are at low risk of severe disease or long-term effects resulting from a specific pathogen.

Prior to the pandemic, the unfortunate reality was that gaining a remedy for vaccine injury, if available at all, was often a Kafkaesque process with a slow and complex route to compensation. Newly published research by an international group of legal and historical scholars shows that official vaccine injury compensation schemes existed in only 25 countries in 2020 (2). Although concerns about rare forms of vaccine adverse effects have led to the near-doubling of international schemes to 43 in 2021, schemes’ performance has been highly uneven. While programs in Nordic countries provide rapid and transparent access to compensation, other schemes erect high hurdles or offer only inadequate redress.

A concerning example is the UK’s Vaccine Damage Payment Scheme (VDPS). Created in 1979, the scheme provides a one-off payment for those who suffer serious disablement because of vaccination. Originally designed as a temporary measure, the VDPS remains on the statute books despite cross-party calls for reform. Criticism has long centred on the fact that the scheme’s payment of £120,000 is far too little for cases of serious injury and is well below comparable damages payments awarded by the courts for such injuries (3). The substantial yet arbitrary hurdle of showing 60% disablement also means that very few payments have actually been made in recent times. The stalling of VDPS payments contrasts sharply with the entirely predictable rise in the number of suspected cases of serious adverse effects reported by patients and medical professionals to the Medicines and Healthcare products Regulatory Agency since the mass-rollout of COVID-19 vaccines (4). Although payments may eventually be made, lack of access to timely and adequate compensation for victims and their families risks leading to legal proceedings, which can damage vaccines reputationally and provide an unwelcome platform for vaccine sceptics.

The UK’s mixed track record with regards to vaccine compensation is far from unique. Many of the world’s poorer countries are unable to compensate victims of adverse effects. The pioneering COVAX No-Fault Compensation Program, which covers 92 low and middle income countries and economies, is attempting to redress this (5). However, the problem is widespread as even high-income countries often provide inadequate support and publish insufficient data to evaluate schemes’ performance. A particular problem remains the opacity of the procedures and the difficulties claimants encounter with access and information.

The global community’s mixed track record in providing for citizens who have behaved altruistically is concerning and contrasts markedly with the extensive legal indemnities and insurance cover provided for vaccine producers. COVID-19 is not over and will not be the last pandemic the world will witness this century. Amidst recent reports of sharp declines of routine vaccine rates, there is an urgent need not only to protect and boost public confidence in COVID-19 vaccines, but also in the many other vaccine regimes we rely on to control diseases. Learning from the past 3 years to create a common best practice framework for vaccine compensation is one way of doing so.

The recent international review of existing vaccine compensation schemes identified four hallmarks signifying best practice across all analysed countries (1): *accessibility*: compensation funds should be accessible, with low legal and financial barriers, good sign-posting, and facilitate the evaluation of harm (2); *transparency*: decision-making processes and compensation frameworks should be transparent with clear funding responsibilities (3); *timeliness*: each scheme should include clear and short time-frames for compensation decision-making (4). Finally, *adequacy* of compensation: the potential significance of vaccine injuries, no matter how rare, should be reflected in compensation that is materially proportionate to the harm suffered.

Legislators around the world would do well to check if their own scheme hits these best practice hallmarks.
